# MOLECULAR BASIS OF CLEFT PALATE IN A SPONTANEOUS DOG MODEL

**DOI:** 10.51894/001c.122868

**Published:** 2024-08-30

**Authors:** Justina Kwaghe, Madelyn Jeffrey, Saira Malik, Brian C. Schutte

**Affiliations:** 1 College of Osteopathic Medicine Michigan State University

## 25

### INTRODUCTION

This ongoing research aims to understand the molecular etiology of the cleft palate observed spontaneously in a colony of dogs. Since palate formation is a highly conserved process across mammals, we expect that the pathophysiological mechanism(s) of cleft palate in this dog family will help us predict and possibly prevent cleft palate in humans.

### OBJECTIVES/HYPOTHESIS

One of the known causes of TTLD is vascular disruption during development. Since numerous dogs were born with both TTLD and Cleft palate, we hypothesis that vascular disruption plays are primary role in both TTLD and Cleft palate. CD31, also known as platelet endothelial cell adhesion molecule 1 (PECAM-1), is a transmembrane glycoprotein expressed by endothelial cells and considered a sensitive and specific marker for vascular differentiation. The objective of this project is to quantify CD31 expression in tissues obtained from fetal pups with cleft versus non-cleft pups.

### METHODS

A series of analysis including western blots was performed on tissues obtained from mouse. These were control experiments to ensure that the antibody used to detect CD31 was specific. Since CD31is highly conserved between all mammalian species, we could then use this antibody to screen for differences in expression of CD31 in puppies born with cleft palate versus without cleft. If the dogs born with the defect have a decrease in CD31 which is required for vascular development, this will support the hypothesis that vascular disruption plays a primary role in Cleft palate.

### RESULTS

After a series of western blots were performed on the mouse tissues, we were able to detect bands of CD31, K17, and beta actin in these tissues successfully. The next step in our research process will be to replicate the same analysis on the dog tissues.

### DISCUSSIONS/CONCLUSION

While there is still a long way to go to reach our objectives and prove the hypothesis of this research, the successful completion of the western blot analysis in mouse tissues is a step in the right direction. With further efforts, the role vascular disruption may play in the alteration of normal palate development resulting in cleft palate will be better understood.

